# Biology and evolving management of resectable dMMR/MSI-H cancers: current status and future perspectives

**DOI:** 10.1007/s00262-025-04223-9

**Published:** 2025-11-06

**Authors:** Tokiyoshi Tanegashima, Masaki Shiota, Kayo Toyosaki, Kouta Funakoshi, Masatoshi Eto

**Affiliations:** 1 Department of Urology, Graduate School of Medical Sciences, Kyushu University, 3-1-1 Maidashi, Higashi-Ku, Fukuoka, 812-8582 Japan; 2 Center for Clinical and Translational Research, Kyushu University Hospital, Fukuoka, Japan

**Keywords:** dMMR, MSI-H, Neoadjuvant immunotherapy, Immune checkpoint inhibitor, Non-operative, Organ preservation

## Abstract

Mismatch repair-deficient (dMMR) or microsatellite instability-high (MSI-H) tumors represent a biologically and immunologically distinct subset of solid malignancies. Defective DNA mismatch repair mechanisms in these tumors lead to the accumulation of insertion-deletion mutations, a hypermutated phenotype, and abundant tumor-specific neoantigens. These features drive robust T cell responses, explaining the remarkable sensitivity of dMMR/MSI-H tumors to immune checkpoint inhibitors (ICIs). Emerging evidence from clinical trials across resectable dMMR/MSI-H colorectal, gastric and gastroesophageal junction, and other solid tumors indicate that ICI therapy can induce profound pathological responses, including high rates of pathological complete response. These responses may permit organ preservation and reduce treatment-associated morbidity, offering a compelling alternative to conventional surgery-based approaches. Despite this promise, several challenges remain. Critical areas warranting further investigation include the refinement of patient selection strategies, clarification of the role of surgery in patients achieving a clinical complete response, and the identification of reliable predictive biomarkers for therapeutic response and resistance. In addition, the long-term oncologic outcomes associated with non-operative management remain to be elucidated. This review comprehensively summarizes the biological basis and emerging clinical evidence for immunotherapy in resectable dMMR/MSI-H solid tumors. We discuss current opportunities and ongoing challenges in refining curative-intent strategies, with the aim of improving outcomes and quality of life for patients with these immunologically distinct cancers.

## Introduction

Mismatch repair-deficient (dMMR) or microsatellite instability-high (MSI-H) tumors comprise a biologically distinct subset of solid malignancies defined by defective DNA repair machinery [1]. This deficiency leads to the accumulation of insertion-deletion mutations, a hypermutated phenotype, and the generation of abundant tumor-specific neoantigens [2, 3]. These characteristics contribute to the high immunogenicity of dMMR/MSI-H tumors and account for their remarkable sensitivity to immune checkpoint inhibitors (ICIs), particularly agents targeting the PD-1/PD-L1 axis.

Initially, this therapeutic vulnerability was demonstrated in the metastatic setting, where ICIs elicited durable responses across various types of dMMR/MSI-H solid tumors [4]. These findings led to the first tissue-agnostic U.S. Food and Drug Administration (FDA) approval of pembrolizumab, marking a milestone in precision oncology. However, as the role of ICIs expands beyond metastatic disease, growing attention has been directed toward their application in earlier, potentially curable stages of cancer. Among these, immunotherapy for early-stage tumors has attracted attention as a promising approach that harnesses the immunologic potential of the tumor microenvironment while enabling organ preservation. Several recent trials have demonstrated that neoadjuvant ICIs can induce high rates of pathological complete response (pCR) and clinical complete response (cCR) in patients with localized dMMR/MSI-H tumors.

In this review, we first provide an overview of the biological features of dMMR/MSI-H tumors that contribute to their heightened sensitivity to ICIs. Subsequently, the latest clinical data on immunotherapy for resectable dMMR/MSI-H solid tumors, along with ongoing clinical trials are summarized. This rapidly evolving field indicates a paradigm shift in the treatment strategy for dMMR/MSI-H tumors—toward immune-based, organ-preserving approaches.

## Biology of dMMR/MSI-H solid tumors

The unique tumor biology of dMMR/MSI-H cancers explain their pronounced sensitivity to immunotherapy. In the following section, we outline the genetic mechanisms, neoantigen generation, and immune evasion strategies that characterize these tumors. This biological foundation provides critical context for interpreting the transformative clinical outcomes observed with ICIs.

### Genetic origins and mechanisms of mismatch repair deficiency

dMMR/MSI-H tumors constitute a biologically distinct subset of malignancies characterized by impaired DNA mismatch repair. This defect arises through either germline mutations in MMR genes such as *MLH1*, *MSH2*, *MSH6*, or *PMS2*, as seen in Lynch syndrome [5], or through sporadic mechanisms, particularly *MLH1* promoter hypermethylation [6]. Biallelic inactivation of these genes is required to disrupt repair activity, resulting in accumulation of replication errors and genomic instability. Tumors with two somatic hits but without germline mutations are referred to as “Lynch-like” tumors [7, 8].

### Mutation burden and histotype-specific profiles

The MMR defect leads to accumulation of insertion–deletion mutations at microsatellite loci and in coding regions, resulting in a hypermutated phenotype. The tumor mutational burden (TMB) differs depending on the specific MMR protein loss and tumor histology. Loss of *MSH2* or *MSH6* generally confers a higher TMB than *MLH1* or *PMS2* deficiency (mean 46.8 vs. 25.0 mut/Mb) [9]. Across histologies, colorectal cancer tends to exhibit the highest TMB, while endometrial and gastric cancers display lower burdens [10]. Each tumor type presents with characteristic mutational landscapes. MSI-H colorectal cancers often harbor *RNF43*, *ARID1A*, *APC*, *KMT2B*, *and KMT2D* mutations, with *BRAF*-V600E being indicative of sporadic etiology [11–13]. MSI-H endometrial cancers are enriched for mutations in *ARID1A*, *PTEN*, *CTCF*, *PIK3CA*, and *JAK1* while MSI-H gastric cancers frequently harbor *ARID1A*, *KMT2D*, *RNF43*, *KMT2B*, and *TP53* mutations [11, 13].

### Neoantigen generation and immune activation

The accumulation of frameshift mutations leads to the production of highly immunogenic neoantigens [14]. These aberrant peptides elicit potent T cell responses, contributing to a microenvironment rich in CD8⁺ cytotoxic T cells and CD4⁺ T helper 1 cells [3]. T cell reactivity toward neoantigens derived from frameshift mutations has been well documented in MSI-H tumors, while that is rarely observed in microsatellite stable or mismatch repair proficient tumors [14]. Lynch syndrome-associated MSI-H tumors tend to have higher TMB, stronger immune infiltration (CD3^⁺^, CD4^⁺^, CD8^⁺^ T cells), and more robust immune recognition than their sporadic counterparts [15, 16]. The immunogenicity of these tumors forms the biological rationale for their exceptional responsiveness to ICIs.

### Mechanisms of immune evasion

Despite their immunogenicity, dMMR/MSI-H tumors frequently evade immune surveillance. These include upregulation of inhibitory checkpoint molecules such as PD-1, PD-L1, CTLA-4, LAG-3, and IDO [3]. Loss-of-function mutations in *B2M*, a component of MHC class I, impair antigen presentation and are enriched in Lynch syndrome-associated tumors [17–19]. Additional immune escape strategies involve mutations in *JAK1*, which abolish IFN-γ responsiveness, and defects in *TAP1*/*TAP2* or HLA genes, all of which further suppress antigen processing [20, 21].

## Recent clinical advances in immunotherapy for resectable dMMR/MSI-H solid tumors

We next review recent clinical trials of ICIs in resectable dMMR/MSI-H tumors (Table 1) and provide the current evidence.

**Table 1:** Overview of immunotherapy trials in patients with resectable dMMR/MSI-H solid tumors

Trial ID	Phase	Patient population	Treatments	Response	trAE ( ≥ grade 3)	Main trAE ( ≥ grade 3)
*Colon/rectal/colorectal cancer*						
NCT03026140 (NICHE)	II	dMMR colon cancer (n = 20)	Neoadjuvant anti-PD-1 antibody nivolumab (2 cycles) + anti-CTLA-4 antibody ipilimumab (1 cycle)	MPR rate: 95%, pCR rate: 60%	13%	Rash, colitis
NCT03026140 (NICHE-2)	II	dMMR colon cancer (n = 111)	Neoadjuvant nivolumab (2 cycles) + ipilimumab (1 cycle)	MPR rate: 95%, pCR rate: 68%	4%	Rash, amylase and lipase increased, myositis, hepatitis
NCT03026140 (NICHE-3)	II	dMMR colon cancer (n = 59)	Neoadjuvant nivolumab (2 cycles) + anti-LAG-3 antibody relatlimab (2 cycles)	MPR rate: 92%, pCR rate: 68%	10%	Hepatitis, colitis, thyroid dysfunction
NCT03926338 (PICC)	II	dMMR/MSI-H colorectal cancer (n = 34)	Neoadjuvant anti-PD-1 antibody toripalimab (6 cycles) with or without celecoxib	Toripalimab with celecoxib pCR rate: 88% Toripalimab without celecoxib pCR rate: 65%	3%	Aspartate aminotransferase increased
NCT04165772	II	dMMR rectal cancer (n = 12)	Anti-PD-1 antibody dostarlimab (6 months)	cCR rate: 100%	0%	NA
NCT04165772 (Cohort 1)	II	dMMR rectal cancer (n = 49)	Dostarlimab (6 months)	cCR rate: 100%	4.8% (Cohort 1 and 2)	Diabetes, lung infection, hypothyroidism, encephalitis, neutropenia, febrile nuetrophenia
NCT05197322 (NEOPRISM-CRC)	II	dMMR/MSI-H colorectal cancer (n = 32)	Neoadjuvant anti-PD-1 antibody pembrolizumab (3 cycles)	pCR rate: 53%	0%	NA
*Gastric/gastroesophageal junction cancer*						
NCT04006262 (NEONIPIGA)	II	dMMR/MSI-H gastric or esophagogastric junction cancer (n = 29)	Neoadjuvant nivolumab (6 cycles) + ipilimumab (2 cycles) follwed by surgery and adjuvant nivolumab (9 cycles)	pCR rate: 58.6%	19%	Colitis/ileitis, hepatitis
NCT04817826 (INFINITY-Cohort 1)	II	dMMR/MSI-H gastric or gastroesophageal junction cancer (n = 15)	Neoadjuvant anti-CTLA-4 antibody tremelimumab (1 cycle) + anti-PD-L1 antibody durvalumab (3 cycle)	MPR rate: 80%, pCR rate: 60%	17%	Colitis, pneumonitis, liver toxicity
NCT04817826 (INFINITY-Cohort 2)	II	dMMR/MSI-H gastric or gastroesophageal junction cancer (n = 17)	Tremelimumab (1 cycle) + durvalumab (3 cycle)	cCR rate: 76.5%	17%	Colitis, γ-glutamyltransferase increased, thyroiditis
*Tumor-agonistic*						
NCT04165772 (Cohort 2)	II	dMMR non-rectal solid tumor (n = 54)	Dostarlimab (6 months)	cCR rate: 65%	4.8% (Cohort 1 and 2)	Diabetes, lung infection, hypothyroidism, encephalitis, neutropenia, febrile nuetrophenia
NCT04082572	II	dMMR/MSI-H colorectal cancer (n = 27) dMMR/MSI-H non-colorectal cancer (n = 8)	Pembrolizumab (6 months before surgery or 12 months)	rCR rate: 30%, rPR rate: 52%	6%	Alanine aminotransferase increased, aspartate aminotransferase increased, hyperglycemia

*dMMR* mismatch repair-deficient, *MSI-H* microsatellite instability-high, *MPR* major pathological response, *pCR* pathological complete response, *cCR* clinical complete response, *rCR* radiographic complete response, *rPR* radiographic partial response, *trAE* treatment-related adverse event

### Colon/rectal/colorectal cancer

Among resectable dMMR/MSI-H tumors, colorectal cancer is the most extensively studied, with increasing evidence supporting the efficacy of neoadjuvant ICIs.

The NICHE trials (NICHE, NICHE-2, and NICHE-3; NCT03026140) have provided compelling evidence for the efficacy and safety of neoadjuvant ICIs in patients with dMMR colon cancer. In the original NICHE trial, patients with resectable colon cancer received two doses of nivolumab (anti–PD-1) and a single dose of ipilimumab (anti–CTLA-4) [22]. Among 20 patients with dMMR tumors, 95% patients achieved a major pathological response (MPR), with 60% attaining a pCR. Building on this, the NICHE-2 trial enrolled 111 patients with locally advanced, non-metastatic dMMR colon cancer [23]. Patients received the same regimen as NICHE: two doses of nivolumab and one dose of ipilimumab. The study reported a 95% MPR rate, including a 68% pCR rate. Treatment-related adverse events (trAEs) were infrequent (4% grade 3–4), and 98% patients underwent timely surgery. No recurrence was observed at a median follow-up of 26 months. The NICHE-3 trial investigated an alternative combination of nivolumab and relatlimab (anti–LAG-3) in 59 patients with resectable dMMR colon cancer [24]. This regimen resulted in a 92% MPR rate, with 68% achieving pCR. Grade 3–4 trAEs occurred in 10% of patients. Notably, even patients who received only a single cycle exhibited robust responses.

A recent single-center, randomised, phase 2 trial (PICC study) evaluated the efficacy and safety of neoadjuvant toripalimab (anti–PD-1) with or without celecoxib in patients with dMMR/MSI-H locally advanced colorectal cancer [25]. A total of 34 patients were randomly assigned to receive either toripalimab monotherapy or toripalimab plus celecoxib for six cycles prior to surgery. All patients received study treatment and underwent surgical resection. 15 of 17 patients (88%) in the toripalimab plus celecoxib group and 11 of 17 patients (65%) in the toripalimab monotherapy group had a pCR. Only one (3%) of 34 patients in the toripalimab plus celecoxib group, had a grade 3 or higher trAE during the neoadjuvant treatment.

A phase 2 trial (NCT04165772) evaluated the efficacy of single-agent dostarlimab (anti–PD-1) in patients with stage II–III dMMR rectal cancer. In this prospective study, patients received dostarlimab every three weeks for 6 months. All 12 patients who completed treatment achieved a cCR, as assessed by magnetic resonance imaging (MRI), positron emission tomography (PET), endoscopy, digital rectal examination, and biopsy, without the need for chemoradiotherapy or surgery. No disease progression was observed during a median follow-up of 12 months [26]. Building upon these promising results, the same trial was subsequently expanded as cohort 1 of the phase 2 study (NCT04165772) to confirm the efficacy and durability of this approach in a larger cohort [27]. A total of 50 patients with stage II–III dMMR rectal cancer were enrolled. Of the 49 patients who completed the planned six-month course of dostarlimab, all achieved a cCR as determined by MRI, endoscopy, digital rectal examination, and biopsy. No patient required chemoradiotherapy or surgery, and no local or distant recurrences were observed at a median follow-up of 30.2 months.

The NEOPRISM-CRC trial (NCT05197322) introduced a tumor mutation burden (TMB)-stratified approach using neoadjuvant pembrolizumab (anti–PD-1) in patients with high-risk stage II–III dMMR/MSI-H colorectal cancer [28]. Patients with TMB-high or -medium tumors received three cycles of pembrolizumab, while those with TMB-low tumors received a single cycle prior to surgery. Among 32 enrolled patients, the intent-to-treat pCR rate was 53%. Notably, most patients were TMB-high, and no grade 3 or higher trAEs or disease recurrences were observed at a median follow-up of 6 months.

Collectively, these studies demonstrate the transformative potential of immunotherapy in resectable dMMR/MSI-H colon/rectal/colorectal cancer, with exceptional response rates, favorable safety profiles, and early signs of durable disease control. In particular, the unprecedented rates of organ preservation and absence of recurrence in dMMR rectal cancer underscore the extraordinary sensitivity of these tumors to ICIs, supporting not only the feasibility of non-operative management but also a potential paradigm shift in curative treatment, preserving both oncologic outcomes and patient quality of life.

### Gastric/gastroesophageal junction cancer

Gastric and gastroesophageal junction (GEJ) cancers with dMMR/MSI-H represent a smaller subset of gastrointestinal malignancies; however, recent trials have begun to reveal the remarkable efficacy of neoadjuvant ICI–based strategies in this population.

The NEONIPIGA study (NCT04006262) was a single-arm, phase II trial evaluating neoadjuvant nivolumab and low-dose ipilimumab, followed by surgery and adjuvant nivolumab in patients with resectable dMMR/MSI-H gastric or GEJ adenocarcinoma [29]. Among 32 enrolled patients, 29 underwent surgery, of whom 58.6% achieved a pCR, and all achieved no residual tumor. The neoadjuvant immunotherapy regimen was well tolerated, with grade 3–4 trAEs observed in 19% of patients. Importantly, no patient experienced relapses at a median follow-up of 14.9 months, and three patients with cCR declined surgery, raising the possibility of a non-operative management strategy.

The INFINITY study (NCT04817826) further investigated another immunotherapy using tremelimumab (anti–CTLA-4) and durvalumab (anti–PD-L1) in two distinct cohorts of patients with dMMR/MSI-H resectable gastric or GEJ cancer [30]. In cohort 1, 15 evaluable patients received neoadjuvant therapy, and 60% achieved pCR, with MPR observed in 80%. However, the pCR rate was significantly lower in T4 tumors (17%) compared to T2–T3 tumors (89%), leading to the exclusion of T4 tumors from cohort 2. At a median follow-up of 28.1 months, the 2-year gastric cancer-specific progression-free and overall survival rates were 85% and 92%, respectively. In cohort 2, the study evaluated a non-operative management approach in patients who achieved cCR after immunotherapy. Of the 17 evaluable patients, 13 (76.5%) achieved cCR and entered non-operative management. At 11.5 months of median follow-up, only one patient experienced local regrowth and underwent successful salvage surgery. The 12-month gastrectomy-free survival rate was 64.2%, demonstrating the feasibility of surgery-sparing strategies in selected patients with dMMR/MSI-H gastric and GEJ cancer.

These findings suggest the potential to reconsider the standard surgical approach, especially in patients with deep responses to neoadjuvant immunotherapy. However, the optimal duration of therapy, identification of reliable predictors of CR, and the determination of which patients still require surgery need to be clarified in future clinical trials.

### Tumor-agnostic trials

Beyond organ-specific studies, several tumor-agnostic trials have explored the use of neoadjuvant ICIs across diverse dMMR/MSI-H solid tumors.

The phase 2 trial (NCT04165772), cohort 2, also investigated the applicability of dostarlimab comprising patients with stage I–III non-rectal dMMR solid tumors amenable to curative surgery [27]. This cohort included tumors of the colon, stomach, GEJ, urothelial tract, hepatobiliary system, and other sites. Among the 54 patients in the non-rectal cohort who completed treatment, 35 (65%) achieved a cCR, and 33 (61%) elected non-operative management. At a median follow-up of 14.9 months, the 2-year recurrence-free survival rate in this cohort was 85%. The option for curative resection was preserved in all patients, and no case progressed to unresectable disease during treatment. The response rates varied by tumor type, with cCR observed in 100% of urothelial and hepatobiliary cancers, 82% of colon, 57% of gastric, and none of endometrial, prostate, or esophageal cancers. Importantly, early clearance of circulating tumor DNA (ctDNA) was strongly correlated with response, supporting its role as a real-time biomarker for treatment monitoring and minimal residual disease detection.

A single-center phase II trial (NCT04082572) aimed to evaluate the safety and efficacy of preoperative pembrolizumab in patients with localized, high-risk resectable or unresectable dMMR/MSI-H solid tumors across multiple organ types [31]. Thirty-five patients with histologically confirmed dMMR/MSI-H cancers were enrolled, including colorectal (77%), pancreatic, duodenal, endometrial, and gastric cancers. Unresectable disease was observed in one-quarter of all patients at the time of presentation. Patients received pembrolizumab for 6 months before elective surgery or 12 months in a non-operative management protocol. Of the 33 evaluable patients, the radiographic objective response rate (ORR) was 82%, with achieving 30% radiographic CR and 52% PR. Endoscopic CR was observed in 63% of evaluable patients. Among the 17 patients who underwent surgical resection, 65% achieved pCR. Notably, 10 patients chose non-operative management and completed 1 year of pembrolizumab without recurrence during the median follow-up of 9.5 months.

These tumor-agnostic trials suggest that neoadjuvant immunotherapy demonstrates high clinical efficacy and the potential for organ preservation across various dMMR/MSI-H solid tumors, while also highlighting the utility of ctDNA as a tool for treatment monitoring.

## Discussion and future perspective

The profound clinical efficacy of ICIs in resectable dMMR/MSI-H solid tumors reflects their unique tumor biology. dMMR/MSI-H tumors harbor defective DNA mismatch repair mechanisms, resulting in widespread frameshift mutations and a hypermutated phenotype. This, in turn, leads to the generation of numerous neoantigens, which are efficiently presented via MHC molecules and elicit potent T cell–mediated immune responses. These features underline the exceptional immunogenicity of dMMR/MSI-H tumors and their sensitivity to ICIs.

Promising results have been reported in the perioperative ICI treatment of resectable dMMR/MSI-H solid tumors, particularly colorectal cancer. In addition, several ongoing clinical trials are still investigating the optimal role of ICIs in the treatment of resectable dMMR/MSI-H solid tumors. These trials aim to shift the treatment paradigm from surgery-centered invasive approaches to immunotherapy-based strategies, as well as to prolong the duration of organ preservation. Table 2 summarizes selected ongoing phase II and III trials evaluating immunotherapy regimens in this setting. The primary endpoints include multiple measures such as pCR, cCR, major clinical response (MCR), and event-free survival (EFS), indicating that therapeutic efficacy in this unique tumor subset is being assessed from multiple perspectives. These continued efforts are expected to redefine the future standard of care for resectable dMMR/MSI-H tumors and to enable surgery-sparing approaches in appropriately selected patients.

**Table 2:** Selected ongoing immunotherapy trials in patients with resectable dMMR/MSI-H solid tumors

Trial	Phase	Patient population	Treatments	Primary outcome	Reference
*Colon/rectal/colorectal cancer*					
NCT04643041 (BASKET)	II	dMMR/MSI-H distal rectal cancer	Anti-PD-1 antibody sintilimab (6 cycles)	1 year DFS rate	https://clinicaltrials.gov/study/NCT04643041
NCT05239546 (NAIO)	II	dMMR colon cancer	Neoadjuvant anti-PD-1 antibody dostarlimab (6 cycles) and adjuvant dostarlimab (8 cycles)	18 weeks MCR rate	https://clinicaltrials.gov/study/NCT05239546
NCT05723562 (AZUR-1)	II	dMMR rectal cancer	Dostarlimab (9 cycles)	1 year cCR rate	https://clinicaltrials.gov/study/NCT05723562
NCT05815290 (NCC3626)	II	dMMR/MSI-H colorectal cancer	Anti-PD-1/CTLA-4 bispecific antibody cadonilimab (8 cycles)	cCR rate	https://clinicaltrials.gov/study/NCT05815290
NCT05855200 (AZUR-2)	III	dMMR/MSI-H colon cancer	Dostarlimab pre- and post-surgery vs. standard of care post surgery (adjuvant FOLFOX/CAPEOX or watch and wait approach)	EFS	https://clinicaltrials.gov/study/NCT05855200
NCT05890742	III	dMMR/MSI-H colon cancer	Neoadjuvant anti-CTLA-4 antibody IBI310 (1 cycle) and sintilimab (2 cycles) vs. radical surgery	pCR rate and EFS	https://clinicaltrials.gov/study/NCT05890742
NCT06205836	II	dMMR/MSI-H colorectal cancer	Anti-PD-1 antibody cemiplimab with or without anti-LAG3 antibody fianlimab (4 cycles)	pCR/cCR rate	https://clinicaltrials.gov/study/NCT06205836
NCT06262581	II	dMMR/MSI-H colorectal cancer	Neoadjuvant anti-PD-1 antibody tisleizumab (4 cycles)	pCR rate	https://clinicaltrials.gov/study/NCT06262581
NCT06477991 (WINDOW)	II	dMMR/MSI-H colorectal cancer	Neoadjuvant tireilizumab (at least 4 cycles)	cCR rate with negative minimal residual disease	https://clinicaltrials.gov/study/NCT06477991
NCT06646445 (PREMICES)	II	dMMR/MSI-H colon cancer	Anti-PD-1 antibody pembrolizumab (8 cycles ± 9 additional cycles) plus watch-and-wait approach vs. surgical resection ± adjuvant chemotherapy (5-fluorouracil/capecitabine ± oxaliplatin 3 months to 6 months)	The rate of success of the experimental strategy at 6 months or after two successive colonoscopies	https://clinicaltrials.gov/study/NCT06646445
NCT06686576	III	dMMR/MSI-H colon cancer	Neoadjuvant and adjuvant anti-PD-1/CTLA-4 bispecific antibody QL1706 vs. surgery with or without adjuvant CAPEOX	pCR rate and EFS	https://clinicaltrials.gov/study/NCT06686576
NCT06698757	II	dMMR/MSI-H colon cancer	Neoadjuvant sintilmab (8 cycles or 4 cycles)	pCR rate	https://clinicaltrials.gov/study/NCT06698757
NCT06903858 (PICC-3)	II	dMMR/MSI-H colorectal cancer	Neoadjuvant anti-PD-1 antibody toripalimab (12 cycles) with celecoxib (6 months)	EFS	https://clinicaltrials.gov/study/NCT06903858
jRCT2031220484 (VOLTAGE-2)	II	dMMR rectal cancer	Anti-PD-1 antibody nivolumab (up to 96 weeks)	2 years cCR rate	https://www.esmogastro.org/article/S2949-8198(23)00046-8/pdf
*Gastric/gastroesophageal junction cancer*					
NCT05836584	II	dMMR/MSI-H gastric or gastroesophageal junction cancer	Neoadjuvant and adjuvant chemotherapy + anti-PD-L1 antibody atezolizumab or atezolizumab alone	EFS	https://clinicaltrials.gov/study/NCT05836584
NCT06059495 (DEWI)	II	dMMR/MSI-H gastric or gastroesophageal junction cancer	Dostarlimab (4 cycles) and adaptive treatment strategy	1 year cCR rate	https://clinicaltrials.gov/study/NCT06059495
NCT06250036 (ZODIAC)	II	dMMR/MSI-H gastric or gastroesophageal junction cancer	Neoadjuvant (4 cycles) and adjuvant (6 cycles) anti-PD-1 antibody zimberelimab + anti-TIGIT antibody domvanalimab or zimberelimab alone	pCR rate	https://clinicaltrials.gov/study/NCT06250036
*Others*					
NCT06279130 (NEOASIS)	II	dMMR solid tumors	Neoadjuvant anti-CTLA-4 antibody botensilimab (1 cycle) + anti-PD-1 antibody balstilimab (2 cycles)	MPR rate	https://clinicaltrials.gov/study/NCT06279130
NCT07013851 (NADIA)	II	dMMR/MSI-H endometrial cancer	Neoadjuvant dostarlimab (4 cycles) and adjuvant chemotherapy including dostarlimab	cCR rate after neoadjuvant dostarlimab	https://clinicaltrials.gov/study/NCT07013851

*dMMR* mismatch repair-deficient, *MSI-H* microsatellite instability-high, *DFS* disease free survival, *MCR* major clinical response, *cCR* clinical complete response, *EFS* event free survival, *pCR* pathological complete response, *MPR* major pathological response

As dMMR/MSI-H is more prevalent in gastrointestinal cancers such as colorectal and gastric cancer [11], numerous clinical trials have been actively conducted in these tumor types. However, even in cancers with a lower prevalence of dMMR/MSI-H, such as urothelial and hepatobiliary cancers, high efficacy of immunotherapy has been demonstrated when dMMR/MSI-H is present [27]. Moreover, in Lynch syndrome, which is a hereditary cancer predisposition syndrome caused by germline mutations in DNA mismatch repair genes and is characterized by the development of malignancies across multiple organs, including the colorectum, endometrium, ovary, stomach, small intestine, urinary tract, and hepatobiliary system, immunotherapy has also been reported to be effective [32].

Current challenges in the management of resectable dMMR/MSI-H tumors include the uncertainty regarding the long-term durability of organ-preserving strategies. Although promising early outcomes have been reported, robust prospective validation is required before these approaches can be widely implemented in clinical practice. Standardizing cCR definitions and surveillance protocols will be essential to ensure the safe expansion of non-operative management. In addition, traditional biomarkers such as PD-L1 expression and TMB have shown limited predictive value, whereas emerging biomarkers—such as early ctDNA clearance and spatial immune architecture—hold promise for identifying patients most likely to benefit.

Alongside these efforts to optimize efficacy and patient selection, evaluating the safety and tolerability of perioperative ICI therapy is also important. In addition to the remarkable efficacy observed across neoadjuvant immunotherapy trials for resectable dMMR/MSI-H tumors, the safety profile has also been generally favorable (Table 1). Grade 3–4 trAEs were reported in 0–19% of patients, including diarrhea/colitis, hepatitis, rash, and endocrinopathies. Notably, severe hematologic toxicities such as febrile neutropenia, which are commonly associated with cytotoxic chemotherapy, were rarely observed. These findings highlight the distinct toxicity spectrum of ICIs compared with conventional perioperative chemotherapy.

Furthermore, the optimal duration, sequencing, and combination strategies for ICIs have not yet been established. The potential synergistic effects of combining ICIs with other modalities, including radiotherapy and targeted agents, also warrant further investigation. Ethical considerations must guide the implementation of non-operative approaches, balancing oncologic safety with patient autonomy and quality of life. Moving forward, clarifying cancer type–specific efficacy and clinical relevance will be critical to refining patient selection and expanding the benefits of immunotherapy across tumor types.

## Conclusions

Immunotherapy for resectable dMMR/MSI-H tumors has demonstrated transformative potential across diverse malignancies. Consistently high pathological response rates, favorable safety profiles, and the increasing feasibility of organ preservation highlight its promise as a paradigm-shifting strategy in precision oncology. As clinical evidence continues to grow, the incorporation of ICIs into early-stage treatment algorithms is poised to redefine the standard of care. Realizing this potential will require addressing key challenges, including optimal patient selection, resistance mechanisms, reliable biomarkers for response monitoring, and long-term oncologic outcomes. The integration of immunotherapy into standard early-stage treatment pathways has the potential to transform surgical oncology—evolving from the treatment aimed solely at cure to the treatment that achieves cure while preserving organs.

## Data Availability

No datasets were generated or analysed during the current study.
